# Pauci-immune Glomerulonephritis in Systemic Lupus Erythematosus (SLE)

**DOI:** 10.7759/cureus.2949

**Published:** 2018-07-09

**Authors:** Jason Liebowitz, Derek Fine, Philip Seo, Michelle Petri, Kirthi Machireddy, Uzma Haque, Rebecca Manno, Homa Timlin

**Affiliations:** 1 Medicine, The Johns Hopkins University School of Medicine, Baltimore, USA; 2 Nephrology, Johns Hopkins Bayview Medical Center, Baltimore, USA; 3 Rheumatology, The Johns Hopkins University School of Medicine, Baltimore, USA; 4 University of the Sciences, Philadelphia, USA; 5 Medicine, The Johns Hopkins University School of Medicine, Essex, USA

**Keywords:** pauci-immune glomerulonephritis, lupus

## Abstract

Glomerulonephritis (GN) in lupus is generally an immune complex glomerulonephritis from the deposition of immunoglobulin and complements. Pauci-immune GN is the most common cause of rapidly progressive GN and is frequently associated with an anti-nuclear cytoplasmic antibody (ANCA). We report a patient with a history of systemic lupus erythematosus who presented with worsening proteinuria and was subsequently diagnosed with pauci-immune GN on renal biopsy, in the absence of ANCA.

## Introduction

Renal involvement in systemic lupus erythematosus (SLE) is usually an immune complex glomerulonephritis. Pauci-immune necrotizing and crescentic glomerulonephritis (NCGN) refers to extensive glomerular inflammation with few or no immune deposits that may result in a rapid decline in renal function. Pauci-immune glomerulonephritis is frequently associated with an anti-nuclear cytoplasmic antibody (ANCA) [1]. An overlap syndrome of lupus and ANCA-associated vasculitis is rare, with an estimated prevalence of 2% [2]. Pauci-immune glomerulonephritis (GN) in lupus was first described in 1983 in four patients who had minimal serologic activity [3]. Subsequent reports of pauci-immune glomerulonephritis have included patients with serologic activity of lupus, positive anti-cardiolipin antibody, and evidence of thrombotic microangiopathy on biopsy, with the interval between diagnosis of SLE and diagnosis of pauci-immune glomerulonephritis ranging from two months to 12 years [4]. Although 15% to 20% of lupus patients have circulating ANCA, these reports included patients who were ANCA-negative [5]. ANCAs appear to inﬂuence the histological pattern of lupus nephritis and are associated with worse baseline renal function and more active lupus serology [6]. We present a patient with SLE who developed proteinuria 13 years after her original diagnosis and underwent renal biopsies that revealed pauci-immune necrotizing and crescentic GN in the setting of negative ANCAs.

## Case presentation

A woman in her late 30s, with a history of SLE characterized by positive anti-nuclear antibodies, anti-Smith antibodies (1:160), anti-double-stranded deoxyribonucleic acid (anti-dsDNA) antibodies (> 1:640), anti-ribonucleoprotein (anti-RNP) antibodies, lupus anticoagulant, immunoglobulin M (IgM) anti-cardiolipin antibodies (27, normal: 0 - 12 U/ml), hypocomplementemia (C3 < 40, normal: 90 - 165 mg/dl; C4 < 8, normal: 10 - 40 mg/dl), rheumatoid factor of 50 IU/mL (negative: < 13.9), elevated erythrocyte sedimentation rate (ESR), arthritis, lymphopenia, and thrombocytopenia, presented to our clinic with new proteinuria (spot urine protein/creatinine ratio 1,059 mg/g) and a normal creatinine (0.6 mg/dl). Evaluation for antibodies of perinuclear (p)-ANCA, cytoplasmic (c)-ANCA, and atypical p-ANCA were all negative. She underwent a renal biopsy that demonstrated focal, mild necrotizing crescentic glomerulonephritis. Glomerular staining showed non-specific 1+ linear staining of the glomerular basement membrane and rare 1+ granular mesangial staining for IgG, IgM, and kappa but with negative staining for immunoglobulin A (IgA), C3, C1q, and lambda. Taken together, this renal biopsy was summarized as a pauci-immune focal necrotizing/extracapillary proliferative glomerulonephritis. The patient was treated with rituximab, mycophenolate mofetil (3g daily), and prednisone but continued to demonstrate proteinuria with 0.73 g/24 hours, creatinine of 0.7 mg/dl, and no hematuria. She underwent a second renal biopsy (at a time when serologies demonstrated low C3 at 77 mg/dl and positive anti-dsDNA antibodies), which resembled class II lupus nephritis with limited subcapsular glomerular sample with deposits staining for IgG, IgM, kappa light chain, and C3, along capillary loops and peripheral mesangium with sparse deposits on electron microscopy. Given the concerns for steroid side effects, a change in insurance, and the lack of follow-ups, she was maintained on mycophenolate mofetil, 3g daily, and a low dose of prednisone, 5 mg, along with lisinopril. However, she continued to experience prolonged morning joint stiffness and her hand MRI demonstrated synovitis. Adding methotrexate led to significant improvement in her arthritis. By 23 months after the initial renal biopsy, she continued to have proteinuria, intermittently low C3, and positive anti-dsDNA antibodies and underwent a third renal biopsy. This biopsy, similar to the first sample, demonstrated a pauci-immune focal proliferative segmental crescentic/necrotizing lesion in 30% of the glomeruli. On repeat serologic testing, her c-ANCAs, p-ANCAs, and atypical ANCAs were still negative.

## Discussion

Pauci-immune glomerulonephritis is a type of rapidly progressive glomerulonephritis which is generally associated with ANCA positive vasculitis, whereas SLE nephritis is known to be due to immune complex and complements deposition giving it a “full house” pattern on immunofluorescence. Pauci-immune glomerulonephritis represents almost 80% of all cases of rapidly progressive glomerulonephritis and its incidence in the United States per year is about 7-10 cases per million [6]. Rapidly progressive glomerulonephritis (RPGN) is classified as pauci-immune glomerulonephritis (PIGN), immune-mediated glomerulonephritis, and anti-glomerular basement membrane (anti-GBM) glomerulonephritis. It is estimated that about 80% of patients with pauci-immune glomerulonephritis have myeloperoxidase (MPO) and p-ANCA, whereas the rest have anti-proteinase 3 (PR3)-cytoplasmic ANCA antibodies (c-ANCA) [7]. Lightstone et al. compared 32 ANCA-positive biopsies to 222 ANCA negative biopsies from patients with lupus nephritis. The majority (82%) of ANCA-positive patients had anti-myeloperoxidase antibodies. Class IV-S lupus nephritis and glomerular necrosis were more common, and isolated Class V lupus nephritis was less common in the ANCA-positive group. ANCA-positive patients had signiﬁcantly higher dsDNA titers, and higher serum creatinine at the time of biopsy [6]. 

## Conclusions

Pauci-immune glomerulonephritis in the background of SLE (with negative ANCA) is an unusual presentation that was seen in our patient. The absence of immune deposits suggests that antibody-independent cell-mediated immunity may mediate pauci-immune GN. Further scientific data is needed to determine the absolute prevalence and pathophysiology of this condition.
